# An Aptamer-Based gFET-Sensor for Specific Quantification of Gene Therapeutic Human Adenovirus Type 5

**DOI:** 10.3390/bios15090605

**Published:** 2025-09-14

**Authors:** Runliu Li, Ann-Kathrin Kissmann, Hu Xing, Roger Hasler, Christoph Kleber, Wolfgang Knoll, Hannes Schmietendorf, Tatjana Engler, Lea Krutzke, Stefan Kochanek, Frank Rosenau

**Affiliations:** 1 Institute of Pharmaceutical Biotechnology, Ulm University, Albert-Einstein-Allee 11, 89081 Ulm, Germany; runliu.li@uni-ulm.de (R.L.); ann-kathrin.kissmann@uni-ulm.de (A.-K.K.); dolphinzyt2418@gmail.com (H.X.); 2 Faculty of Medicine and Dentistry, Danube Private University, Steiner Landstraße 124, 3500 Krems an der Donau, Austria; roger.hasler@dp-uni.ac.at (R.H.); christoph.kleber@dp-uni.ac.at (C.K.); wolfgang.knoll@dp-uni.ac.at (W.K.); 3 Department of Gene Therapy, Ulm University, Helmholtzstraße 8/1, 89081 Ulm, Germany; hannes.schmietendorf@uni-ulm.de (H.S.); tatjana.engler@uni-ulm.de (T.E.); lea.krutzke@gmx.de (L.K.); stefan.kochanek@uni-ulm.de (S.K.)

**Keywords:** aptasensor, SELEX, rGO-FET, adenovirus, vector, capsid, protein

## Abstract

The combination of rGO-FETs (reduced Graphene Oxide Field-Effect Transistors) and DNA-oligonucleotide aptamers to sense analytes has been shown to be a promising technological approach, achieving high sensitivity and selectivity. With human adenovirus type 5 (HAdV-5) particles as the target, we here demonstrate the application of the aptamer/FET combination for detection of this medically and biotechnologically relevant viral vector. A focused anti-HAdV-5 aptamer library was evolved in a nine-round SELEX process, allowing for the specific fluorescent labeling of HAdV-5 and related subtypes. Moreover, this library was already sufficient to serve as the binding entity on a gFET sensor for sensitive quantification of the virus particles. Adenoviruses have been widely used as gene delivery vectors for gene therapy and genetic vaccination. The use of adenoviral vectors within the vaccination campaign against COVID-19 emphasized the need for robust biotechnological production processes, which additionally require sensitive product formation monitoring. We believe that these type of gFET-based aptasensors can serve as the technological monitoring basis in virus production processes in the near future.

## 1. Introduction

**Figure 1:**
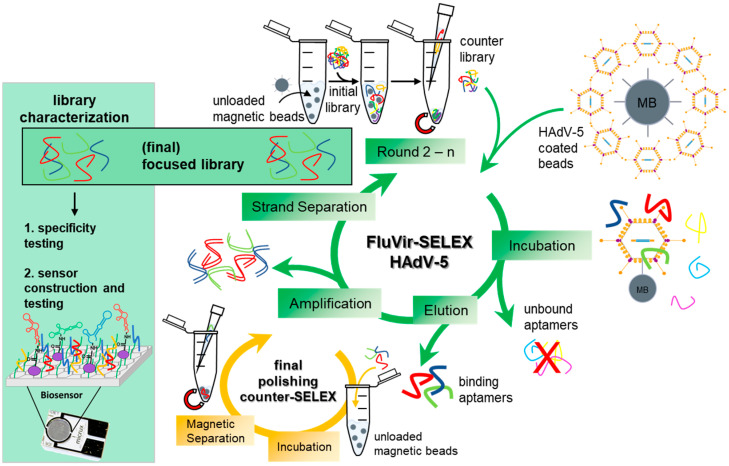
Schematic overview of FluVir-SELEX-based evolution of focused HAdV-5 aptamer library. Initial counter-selection by incubation of an initial aptamer library (~10^12^ individual aptamers), containing 40 randomized nucleotides flanked by two primer binding sites (23 nt each), with unloaded NHS-activated magnetic beads (MB), resulting in an aptamer “counter library” with reduced ap-tamer amounts with specificity against the carrier material. Selection of focused polyclonal aptamer libraries using SELEX against HAdV-5 through reduction in sequence diversity by incubating the counter-selected aptamer library first with unloaded beads and subsequently with the HadV-5 loaded beads. Aptamers with an adequate three-dimensional structure bind to the target, whereas the remaining unbound aptamers are subsequently removed. The bound aptamers are then eluted from the virus by heat denaturation, amplified by PCR, and the unwanted complementary strands are removed prior to the following SELEX round. Using a final counter-selection for aptamer library polishing with unloaded beads, remaining unspecific molecules are removed. The final focused aptamer library is then characterized for affinity and specificity and is used as binding entity for biosensing applications.

Starting with the pioneering work of the Szostak and Gold groups [1,2] introducing nucleic acid based affinity molecules in the year 1990, more than 30 years of intensive (fundamental) research on “aptamers” have relieved today’s scientists of the need to carry out and demonstrate studies on the basic suitability of this class of molecules; rather, the concept of their use has reached an advanced technology level to enable real applications in diagnostics and (pharmaceutical) biotechnology. The methodological essential concept behind a typical aptamer project is the combined evolution and selection of effective DNA or RNA oligonucleotides (“aptamers”) with the desired affinity and specificity towards the respective target [3,4,5,6,7]. Today, many variants of this SELEX process (“Systematic Evolution of Ligands by EXponential enrichment”) exist with technological adaptations for different classes of targets ranging from small molecules, proteins, and whole cells to tissues of the human or animal body or plants [8,9,10,11,12,13,14,15]. With the introduction of the so-called FluMag-SELEX [16], considerable experimental facilitation became available, which made the success of the process monitorable and measurable in a step-by-step fashion, and thus enabled attractive options for fast and efficient optimization measures within the process. This SELEX variant was based on the use of fluorescently (“Flu…”) labeled aptamers throughout the process, which led to the labeling of the respective target immobilized on magnetic particles (“…Mag”) by the freshly isolated sub-library after each round, thereby allowing direct quantitative measurements in fluorimeters or in fluorescence microscopy [16]. Regarding living cells, bacteria, fungi, or human cells as particles with the convenient opportunity to separate them simply by centrifugation, we have introduced—inspired by the FluMag-strategy—the “FluCell”-SELEX, and for plant tissues, the “FluRoot”-SELEX, which allowed the efficient and easy monitoring of SELEX processes against a number of pathogenic and probiotic bacteria [17,18,19,20,21,22], the development of aptamer libraries for the discrimination of human cells from major pathogenic yeast cells [23,24], and of libraries for the discrimination of developmental zones of the roots of the model plants *Arabidopsis thaliana* and *Hordeum vulgare* [25]. Here, we show, as an adaptation of the FluMag-SELEX, the functionality of this concept for the selection of specifically binding aptamers to human adenovirus type 5 (HAdV-5), immobilized on magnetic beads as the carrier material, which we here, consequently, call the “FluVir-SELEX” (Figure 1).

Adenoviruses are important infectious agents commonly causing different diseases of varying severity, for example, of the respiratory tract, the eye, or the gastrointestinal tract, but also causing systemic diseases in immunocompromised patients. As replication-competent (oncolytic) viruses, either alone or in combination with immunostimulatory strategies, they are under clinical development for cancer therapy [26,27]. Replication deficient adenoviral vectors [27] are characterized by efficient gene transfer to many cell types in vitro and in vivo, the large transport capacity for expressed transgenes, the possibility of production at an industrial scale [28], and an overall good safety record. The strong immunogenicity of adenoviruses renders this vector type particularly suitable for genetic vaccination, as has been successfully demonstrated during the vaccination campaign against COVID-19, with approval of three adenovirus-based vaccines based on Chimpanzee Adenovirus Y25 [29] (ChAdOx1-S), human adenovirus type 26 [30] (Ad26.COV2.S), or hAdV-5 [31] (Ad5-nCOV). In this context, the need for the use of specific and sensitive monitoring technologies for the detection of impurities derived from the production cells has recently been shown [32]. Currently, routine diagnostic detection of disease-causing adenovirus infection is based on two PCRs on adenoviral genomic DNA isolated from viral particles: the first for the general detection of adenovirus DNA, and the second for the determination of the specific adenovirus type, both procedures together taking several days [33,34].

EG-FETs (electrolyte-gate Field-Effect Transistors) have become a highly effective platform for biosensing applications [35,36,37], offering advantages including high sensitivity, label-free detection, and the potential for miniaturization and point-of-care diagnostics [38]. They function by modulating channel conductivity through the application of a gate voltage, similar to metal oxide FETs [39,40,41,42]. In this set up, the source and drain electrodes are separated by a semiconductor channel and controlled by a gate electrode. Varying the gate voltage alters the channel’s conductivity, which in turn changes the measured current. Likewise, the binding of biomolecules affects the local electrostatic environment, altering the charge carrier distribution and resulting in a shift in the source–drain current (I_DS_) [43]. By monitoring I_DS_ at a fixed gate voltage, binding events can be detected. Recent research has highlighted the versatility of EG-FETs, particularly when combined with recognition elements including antisense oligonucleotide probes, antibodies, and aptamers [44,45] for detecting a wide range of analytes—from small molecules and proteins to whole bacterial cells [46,47,48,49,50,51].

In this study, we show that the intended expansion of the portfolio for fluorescence-assisted SELEX processes was fully functional for the isolation of a focused aptamer library against HAdV-5. This library was characterized and shown to be specific in fluorescence-based binding assays, similar to the selection steps during the SELEX, and showed specificity, which was also confirmed in magnetic bead-independent dot blots. The library allowed for the discrimination of HAdV-5 from adeno-associated virus type 8 (AAV8), a representative of a different virus family, whereas, as expected, it allowed for the detection of a selection of 12 different adenovirus types. We also show that the combined aptamer and gFET (graphene Field-Effect Transistor) technologies were fully suited to quantitatively measure HAdV-5 dilutions down to 1E4 viral particles per mL, whereas it could perfectly discriminate AAV8 as a control virus. We believe that the choice of the focused aptamer library evolved in this study, and its performance in the experiments with the laboratory set up will pave the way for the development of gFET-based aptasensors for the routine quantification not only of adenoviruses but also of other viruses in diagnostics and for quantitative monitoring in the biotechnological production of viruses and viral vectors. Additionally, this work may inspire researchers from these different fields to advance aptasensor technology, including the creation of multiplexed sensors for future point-of-care applications in diagnostics.

## 2. Materials and Methods

### 2.1. Cell Lines

Cell lines were split twice a week and incubated at a relative humidity of 90%, 37 °C, and 5% CO_2_. N52.E6 cells (split rate 1:3) were cultivated in α-MEM medium (ThermoFisher, Waltham, MA, USA, #22561-021), and A549 cells (ATCC: CCL-185; split rate 1:8; ATCC, Manassas, VA, USA) were cultivated in MEM medium (ThermoFisher, Waltham, MA, USA, #31095-029). Media were supplemented with 10% FCS and 1× penicillin/streptomycin/glutamine (ThermoFisher, Waltham, MA, USA, # 10378-016).

### 2.2. Adenoviral Particles

Human adenovirus type 5 particles (HAdV-5) used in this study were *E1*-deleted, replication-incompetent vectors based on NCBI Reference Sequence AY33986 ∆nt441-3521. A human CMV promoter-driven enhanced GFP expression cassette subcloned from peGFP-N1 plasmid (Takara Bio USA, Inc., San Jose, CA, USA # 6085-1) was introduced at position nt441. All other human adenovirus types used in this study were unmodified replication-competent, wild-type adenoviruses based on NCBI Reference Sequence DQ086466.1 (HAdV-3), NCBI Reference Sequence AY163756.1 (HAdV-11), NCBI Reference Sequence JN226751.1 (HAdV-24), NCBI Reference Sequence EF153474.1 (HAdV-26), NCBI Reference Sequence JN226754.1 (HAdV-29), NCBI Reference Sequence KF268196.1 (HAdV-34), NCBI Reference Sequence AC_000019.1 (HAdV-35), NCBI Reference Sequence AB448778.1 (HAdV-37), NCBI Reference Sequence JN226759 (HAdV-38), NCBI Reference Sequence JN226762.1 (HAdV-43), NCBI Reference Sequence EF153473.1 (HAdV-48), and NCBI Reference Sequence AY737798.1 (HAdV-50).

Replication-incompetent vectors were propagated using *E1*-complementing N52.E6 cells, while replication-competent viruses were produced in A549 cells. The 4E8 cells were transduced with 300 particles/cell and incubated for 48−72 h. Cells were harvested when they started to detach, resuspended in resuspension buffer (50 mM HEPES, 150 mM NaCl, and pH7.4), and lysed by three freeze–thawing cycles. Cell debris was removed by centrifugation at 2000× *g* for 10 min, and particles were purified from cell lysates by two consecutive CsCl density gradient ultracentrifugation steps at 176,000× *g*. Subsequently, particle solutions were desalted by size exclusion chromatography and purified particles were dissolved in 50 mM HEPES, 150 mM NaCl, 10% glycerol, and pH7.4 and stored at −80 °C (PMID: 35402633). Total particle titers were determined by optical density measurements, as previously described. All titers ranged between 7E8 VP/µL to 4E9 VP/µL.

### 2.3. Immobilization of Adenoviral Particles on NHS-Activated Magnetic Beads

A total of 25 µL (for evaluation of coupling efficiencies, 10 µL) NHS-activated magnetic beads (ThermoFisher, Waltham, MA, USA, #88826, concentration: ~10 µg/µL) were placed on a magnetic stand, and the storage buffer was removed. Beads were resuspended in 1 mL of 1 mM HCl and incubated for 1 min before they were washed twice with 1 mL coating buffer (50 mM HEPES, 150 mM NaCl, and pH 7.4). Subsequently, beads were mixed with 1E9 VP/µL bead (referring to a protein amount/mg bead of approximately 25.6 µg of vector or virus particles dissolved in coating buffer in a total volume of 20 µL/µL bead (sample: Input). Beads and particles were incubated overnight at 4 °C, rotating. The next day, supernatant containing unbound particles was removed (sample: FlowThrough FT), and beads were washed twice with 1 mL coating buffer (first wash sample: Wash I). To saturated remaining reactive NHS groups, beads were quenched with 1 mL Ethanolamine (3 M, pH 7.4) for 1 h at room temperature, rotating. Subsequently, beads were washed again three times with 1 mL coating buffer (last wash sample: Wash II). Finally, beads were resuspended in 1 µL coating buffer/µL bead. For the evaluation of coating efficiencies, bead-bound particles were eluted either by heating samples for 5 min at 96 °C (sample: Elution 1) or by supplementation of 1 × SDS loading buffer (10×: 3.5 M β-mercaptoethanol, 625 mM Tris pH 7.5, 20% SDS, 20% glycerol, and bromophenol blue) and heating for 15 min at 96 °C (sample: Elution 2).

### 2.4. SDS-PAGE and Silver Staining

For the evaluation of coating efficiencies, 30 µL of Input, FlowThough (FT), Wash I, and Wash II and 10 µL of Elution 1 were supplemented with 1 × SDS loading buffer. Addition of 1 × SDS loading buffer to 10 µL Elution 2 was omitted since it was already applied during elution. All samples were heated for 5 min at 96 °C. Proteins were separated under reducing conditions by SDS-PAGE (5% stacking/8% separation gels; running buffer: 25 mM Tris, 192 mM Glycine, and 0.1% SDS). Subsequently, proteins were stained by silver staining, as previously described.

### 2.5. In Vitro Selection of Aptamer Libraries Against HAdV-5

A commercial aptamer library (TriLink BioTechnologies, Inc., San Diego, CA, USA) containing approximately 10^15^ unique sequences was used. Each aptamer consisted of a central 40-nucleotide (nt) randomized region flanked by two 23-nt primer binding sites. Aptamer selection was performed using the following primers: a Cyanine 5-labeled forward primer (Cy5 FW: 5′-[Cy5] TAGGGAAGAGAAGGACATATGAT-3′) and a phosphate-labeled reverse primer (Phosphate RW: 5′-[Phosphate] TCAGTGTCATGTACTAGTCAA-3′), both obtained from Biomers.net GmbH (Ulm, Germany). For initial counter-selection, 0.5 mg of empty Pierce™ NHS-activated magnetic beads were washed three times with 500 μL of HEPES buffer (50 mM HEPES, 150 mM NaCl, and pH 7.4). To activate the aptamer library, the single-stranded DNA (ssDNA) was denatured by heating to 95 °C for 5 min, quickly cooled to 4 °C for 5 min, and then incubated at 25 °C for 20 min. The magnetic beads were then suspended in 500 μL HEPES buffer containing 100 pmol of the ssDNA library and incubated at 25 °C for 30 min under gentle rotation in the dark. The unbound aptamers in the supernatant were mixed with 600 pmol of BSA (100 mg/mL) and 600 pmol of tRNA (10 mg/mL) (Table 1) for blocking, and the mixture was then incubated with magnetic beads pre-loaded with HAdV-5. After incubation, unbound aptamers were removed through a washing step with 500 μL of HEPES buffer. Bound aptamers were eluted by heating the beads in 100 μL of 1 × DPBS at 95 °C for 5 min, followed by magnetic separation for 2 min. Eluted aptamers were amplified using emulsion PCR (ePCR). The aqueous phase (200 μL) consisted of 40 μL of 5 × Herculase II reaction buffer, 5 μL dNTP mix (10 mM; 2.5 mM each), 0.5 μL of each primer (Cy5 Forward and Phosphate Reverse), 1 μL of Herculase II Fusion DNA Polymerase (Agilent Technologies, Santa Clara, CA, USA), 113 μL HPLC-grade water, and 20 μL ssDNA. This was emulsified with 400 μL of an oil–surfactant mixture (4.5% Span^®^ 80, 0.4% Tween^®^ 80, 0.05% Triton X-100, and 95.05% mineral oil) using vortex mixing for 10 min. Aliquots (50 μL) of the emulsion were transferred to PCR tubes and amplified under the following conditions: 95 °C for 2 min; 25 cycles of 95 °C for 30 s, 56 °C for 30 s, and 72 °C for 10 s; and followed by a final extension at 72 °C for 2 min. Post-PCR, emulsions were pooled into 2 mL tubes, mixed with 1 mL of isobutanol, centrifuged at 13,000 rpm for 1 min at 25 °C, and the upper oil phase was discarded. DNA products were purified using a commercial kit (MACHEREY-NAGEL GmbH & Co. KG, Düren, Germany) and analyzed on a 2% agarose gel. To obtain ssDNA, the complementary strand was digested using Lambda exonuclease (New England Biolabs, Ipswich, MA, USA). Enzyme inactivation was achieved by heating the mixture at 80 °C for 10 min, followed by further purification using an optimized PCR purification kit (MACHEREY-NAGEL GmbH & Co. KG, Düren, Germany). When needed, isopropanol and 3 M sodium acetate (pH 5) were added to improve DNA recovery. The ssDNA concentration was quantified using a NanoPhotometer (IMPLEN, Munich, Germany). For subsequent SELEX rounds, 10 pmol of ssDNA was used in rounds 2–4, 1 pmol in rounds 5–8, and 0.1 pmol in round 9. Each round began with counter-selection using unloaded beads, followed by target binding with increasing concentrations of BSA and tRNA. From round 4 onward, the washing buffer also contained BSA. After round 9, additional polishing counter-SELEX steps were performed (Table 1), involving incubation of the ssDNA library with empty beads under rotation at 25 °C in the dark, followed by collection of the supernatant containing unbound aptamers. The selection conditions were adapted from our previous successful SELEX processes [52].

### 2.6. rGO-FET-Based Quantitative Measurements

The gFET sensors with gold surfaces ([ED-IDE1-Au], Micrux Technologies, Gijón, Spain) were coated with reduced Graphene Oxide following a previously established protocol [53,54]. In brief, the chips were inspected first under a light microscope to exclude defective devices and were handled exclusively with plastic tweezers to avoid contamination of the interdigitated electrode area. Prior to use, chips were cleaned by sequential sonication in 2% (*v/v*) Hellmanex III (Merck KGaA, Darmstadt, Germany) solution and absolute ethanol, each for 15 min, followed by thorough rinsing with ultrapure water and drying under N_2_. For surface functionalization, the cleaned chips were incubated in a 2% (*v/v*) APTES solution in ethanol for 1 h at room temperature under light protection, rinsed repeatedly with ethanol, and cured at 120 °C for 1 h. Subsequently, Graphene Oxide (GO) was deposited onto the sensing area by spin-coating a 1:5 diluted GO dispersion (70 µL, 1800 rpm, and 60 s), followed by rinsing with water and drying under N_2_. Chemical reduction in the GO was performed by exposing the chips to hydrazine vapor at 60 °C overnight, followed by ventilation at room temperature to remove residual vapor. An additional thermal reduction step was carried out at 200 °C for 2 h. After cooling to room temperature, the chips were stored under vacuum until further use. For functionalization, a solution containing 500 μM PyPEG (polyethylene glycol linked to pyrene) and 50 μM 1-pyrenecarboxylic acid (PCA) in DMSO was prepared. The gFETs were immersed in this mixture and incubated at room temperature in the dark for 12 h. Afterward, the devices were rinsed twice with 1 mL of isopropanol, gently dried under nitrogen, and placed into Micrux All-in-One platform flow chambers, with an Ag/AgCl electrode serving as the gate. To monitor bio-recognition, transfer characteristics (I_DS_V_G_) were recorded using a Keysight U2722A modular source/measure unit (Keysight Technologies, Santa Rosa, CA, USA) and custom LabView-based software developed by our group (National Instruments, Austin, TX, USA). Measurements were performed with a gate voltage sweep from −0.5 V to +0.5 V at a scan rate of 20 mV/s and a fixed drain-source voltage (V_DS_) of 50 mV. Initially, the chip surface was rinsed with distilled water (0.2 mL/min, 10 min), followed by DPBS (0.5 mL/min, 2 min), and then I_DS_V_G_ curves were recorded in buffer. For aptamer immobilization, 1 mL of freshly prepared DPBS containing 15 mM EDC and 15 mM NHS was introduced at a flow rate of 0.2 mL/min for 30 min to activate the carboxyl groups on the surface. The system was then rinsed with DPBS at 0.5 mL/min for 1 min, followed by 10 min at 0.2 mL/min to ensure complete removal of residual EDC and NHS. Next, 1 mL of 1 × DPBS containing 100 pmol of amino-modified aptamers was circulated over the surface at 0.2 mL/min for 40 min to facilitate aptamer attachment. Subsequent I_DS_V_G_ measurements were performed to confirm successful layer-by-layer functionalization prior to the respective individual quantitative sensor measurements for each used sensor chip [53]. Finally, the target HAdV-5 at desired virus concentration in 1 mL of 1 × DPBS was applied to the chip surface for 15 min. Afterwards, the system was washed with DPBS at a flow rate of 0.2 mL/min for 5 min to remove unbound HAdV-5 and then binding was measured by recording the I_DS_V_G_ curves, as described above. Negative control measurements were conducted with AAV8 viral particles.

### 2.7. Dot-Blot Analysis of Aptamer-Binding to Adenoviral Particles

Binding of an HAdV-5 aptamer library to viral particles under native conditions was investigated using a dot-blot procedure. A total of 5E9 HAdV virus particles (VP) or 3E12 adeno-associated virus (AAV) serotype 8 particles (AAV8) were diluted in 20 µL Dulbecco’s phosphate-buffered saline (DPBS) (without Ca^2+^/Mg^2+^) and transferred under vacuum to a nitrocellulose membrane (Cytiva, Amersham^TM^ Protran^TM^ Premium 0.45 µm NC) in a dot-blotting device (S&S MINIFOLD, SPOT-BLOTTER). After drying, the membrane proteins were stained with Ponceau S red solution (SIGMA, St. Louis, MO, USA) to confirm about equal amounts of protein blotted as VPs to the membrane. The membrane was blocked overnight at 4 °C in blocking buffer (50 mM HEPES, 150 mM NaCl, 5% BSA (*w/v*), and 0.05% Tween^®^ 20 (*w/v*)). Biotin-conjugated aptamer libraries against HAdV-5 and apo-RBP4 [46] as the control, respectively, were activated, as described above. A total of 0.5 pmol of the respective aptamer libraries or of a monoclonal mouse-α-Hexon primary antibody (abcam, #ab7428, lot#GR3372598-3), broadly recognizing all HAdV types, was diluted in 3 mL assay buffer (50 mM HEPES, 150 mM NaCl, and 0.05% Tween20 (*w/v*)) and were then incubated with membranes for 1.5 h at room temperature while rotating. Membranes were washed three times for 5 min in assay buffer and then incubated for 1 h at room temperature with streptavidin–horse radish peroxidase (Dako, #P0397, lot#00062147), diluted 1:5000, and a rat-α-mouse-IgG (whole molecule)-peroxidase (SIGMA, #A9044-2ML, lot#031M4752) secondary antibody, diluted 1:20,000 in assay buffer, while rotating. Membranes were washed three times for 5 min in assay buffer. Subsequently, membranes were bathed in developer solution (ThermoScientific, SuperSignal^TM^ West Pico PLUS) for 1 min and dried. Inside a dark chamber, an X-ray film (advansta, LucentBlue X-ray film) was applied to the membrane, exposed for 20 s to chemiluminescence, and developed in an AGFA CP10300 machine (AGFA, Mortsel, Belgium).

## 3. Results

**Figure 2:**
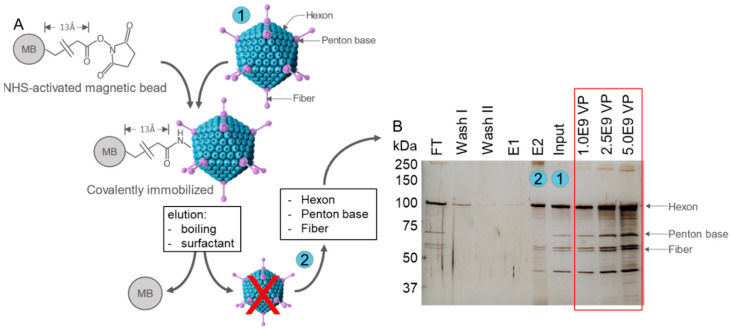
Covalent coupling of HAdV-5 vector particles to NHS-activated beads. (**A**) Schematic illustration of the attachment of HAdV-5 vector particles to NHS-activated magnetic beads (MB) via the NH_2_ groups of surface-exposed capsid proteins Hexon, Fiber, and Penton bases, forming covalent amid bonds and being separated by a short 13Å linker. (**B**) 1E9 HAdV-5 vector particles/µL bead in a total volume of 20 µL/µL bead were covalently attached to NHS-activated magnetic beads. Loading volumes: 30 µL of Input, flow-through (FT), Wash I, and Wash II; 10 µL of Elution 1 (E1) and Elution 2 (E2). Additionally, three defined amounts of purified vector particles (1E9, 2.5E9, and 5E9) were loaded as a reference to estimate vector amounts in the different samples. Viral proteins were separated under reducing conditions by SDS-PAGE and visualized by silver staining. kDa: kilo Dalton.

Adenoviruses (Ad) are non-enveloped, double-stranded DNA viruses with an icosahedral capsid consisting of a Hexon, Fiber, and Penton base as the main solvent-exposed capsid proteins [55]. Ad vector particles were covalently coupled to NHS-activated magnetic beads via the primary amines of the capsid proteins (Figure 2A). Coupling of particles was analyzed by the separation of heat-denatured and reduced samples by SDS-PAGE and the subsequent silver staining of proteins (Figure 2B). Results show comparable amounts of viral proteins in the flow-through (FT) as in the Input sample, confirming the saturation of beads. Vector particles did neither elute during washing nor when particles were only briefly heated (E1). Only when elution was performed by intense heating and in the presence of the SDS loading buffer, containing a detergent and reducing agent (E2), bound viral proteins were released from the beads, which confirmed the covalent attachment of the particles. Three defined reference vector particle (VP) amounts of 1E9, 2.5E9, and 5E9 VP were loaded in parallel. Based on the comparison of protein staining intensities, the amount of bead-bound and eluted particles in E2 was estimated to be ~1E8 VP/µL bead, referring to ~1E7 VP/mg bead or ~2.5 ng/mg bead, given that one VP consists of ~2.56E−10 µg protein.

**Figure 3:**
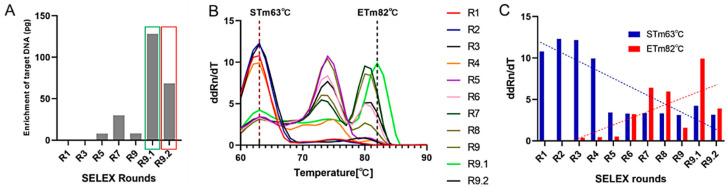
Quantitative PCR-based evolution analysis of anti-HAdV-5 aptamer libraries. (**A**) Melting curves of HAdV-5 aptamer libraries from rounds 1 to 9.1, with temperature peaks at S_Tm_ (63 °C) at the first round of the SELEX process and E_Tm_ (82 °C) at the end of the process. (**B**) Peak shifting analyses for S_Tm_ and E_Tm_. Linear regression analysis was performed to test the relationship between ddRn/dT (change in fluorescence divided by change in temperature) and the different SELEX rounds. (**C**) Enrichment of DNA amount after elution over the SELEX process with blue dashed lines indicating decreasing S_Tm_ and red dashed lines indicating increasing E_Tm_. All experiments were performed in triplicate (n = 3), analyzed using qTOWER^3^G Touch (Analytik Jena GmbH, Jena, Germany), and the fluorescent dye SYBR Green I (Sigma-Aldrich, St. Louis, MO, USA).

In total, nine rounds of selection during iterative FluVir-SELEX were performed, during which the amount of aptamers added was continuously decreased, while the concentrations of BSA and tRNA for blocking and the number of washing steps were continuously increased to select high affinity binding aptamers (Table 1). Additionally, a counter-selection step was conducted using empty (i.e., unloaded) magnetic beads to remove unspecific background bead-binding and, thus, non-HAdV-5-specific aptamers prior to each true selection round to further increase specificity. Following the ninth round, two subsequent final polishing counter-SELEX steps with only unloaded beads were performed to remove residual bead-binding aptamers, delivering the final aptamer libraries 9.1 and 9.2. To monitor the progress of the aptamer enrichment of HAdV-5-binders, a quantitative PCR (qPCR) was performed after each SELEX round, a concept we have introduced as the IMPATIENT-qPCR (“meltIng-teMPerATure-shIft based Evolution moNiToring”) [52]. The amount of eluted aptamers increased throughout the process, suggesting that the desired enrichment of HAdV-5-binding aptamers with library 9.1 was the best, and the “draw-back” round 9.2 indicating the success of the polishing step (Figure 3A). Consequently, the melting temperatures (T_m_) of aptamer libraries increased with the SELEX proceeding to success and, in addition, also reflected the draw back in round 9.2. In general, in this type of experiment, Tm represents the average melting temperature, with S_Tm_ and E_Tm_ being the determined by Tm at the start (round R1) and end of the SELEX (round R9.1), respectively. During the SELEX process, the library melting temperatures shifted from 63 °C (S_Tm_) to 82 °C (E_Tm_) (Figure 3B), indicating the expected development of the library sequence space to more pronounced GC contents, which can be interpreted as the evolution of favorable secondary structures and thus stability and, in turn, with an enhanced affinity to the target epitopes due to a higher number of available potential hydrogen bonds [52]. To compare the shift in the melting curve over the 9 + 2 rounds of SELEX, the relative ddRn/dT values for each round at the peak temperatures of S_Tm_ (R1) and E_Tm_ (R9.1) were quantified, where the ddRn/dT ratios gradually decreased at S_Tm_ and increased at E_Tm_ (Figure 3C).

**Figure 4:**
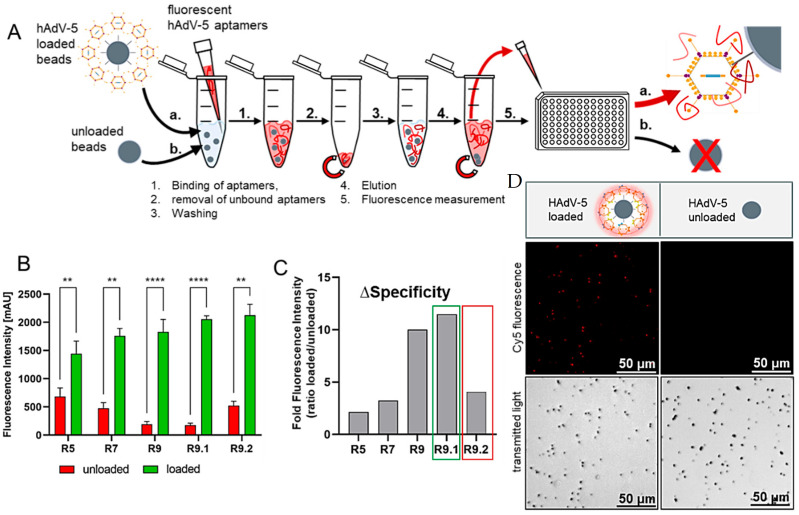
Fluorescence-based aptamer specificity analyses. (**A**) Schematic overview of binding assay with Cy5-labeled anti-HAdV-5 aptamer libraries. The fluorescently labeled aptamers of rounds 5, 7, 9, 9.1, and 9.2 were either incubated with HAdV-5-loaded (a.) or unloaded (b.) magnetic beads (negative control). Beads were washed, bound aptamers eluted by heat (95 °C for 5 min), and quantified by fluorescence measurements. (**B**) Increased affinities and specificities of aptamer libraries towards HAdV-5-loaded beads over the analyzed SELEX rounds. The fluorescence intensity can be correlated with the affinity of the aptamers against the given target. Results are given as mean arbitrary units (mAU) + standard deviation of experiments conducted in triplicate (n = 3), with *p* values < 0.05 considered significant. ** denotes *p* < 0.01 and **** denotes *p* < 0.0001. (**C**) Specificity increase in each round (∆specificity) given as ratio of fluorescence units of aptamers bound to loaded and unloaded beads for each SELEX round. (**D**) Fluorescence microscopy of HAdV-5-loaded beads and unloaded beads after co-incubation with Cy5-labeled aptamer library R9.1 Images were monitored using a Leica DMi8 coded (Leica Microsystems CMS GmbH, Wetzlar, Germany) at ×20 under transmitted light and with Y5 filter.

Subsequently, defined amounts of fluorescently labeled (Cy5) aptamer libraries (5 pmol) from SELEX round 5, 7, and 9 and both polishing rounds 9.1 and 9.2, were incubated with unloaded beads and HAdV-5-loaded magnetic beads for 30 min at 25 °C. Bound aptamers were eluted, and the fluorescence intensity of the samples derived from unloaded or HAdV-5-loaded beads were compared (Figure 4A). The specific signals delivered by the aptamer libraries increased for the HAdV-5-loaded beads over the course of the process, with the non-specific signals resulting from samples containing empty beads decreasing and round 9.1 representing the maximum (specific signal) as well as the minimum (unspecific signal) (Figure 4B). As a consequence, the ratios of the signals of loaded to unloaded (loaded: unloaded) beads reached their maximum in round 9.1 (Figure 4C). In addition, the final aptamer library R9.1 was suited to fluorescently label the virus-covered magnetic particles and allowed for their discrimination from empty control beads by fluorescence microscopy (Figure 4D).

**Figure 5:**
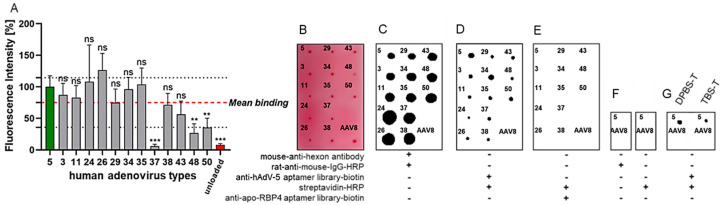
Cy5-labeled HAdV-5-R9.1 aptamer library was co-incubated with unloaded beads or beads loaded with HAdV-5 or the human adenoviruses HAdV-3, HAdV-11, HAdV-24, HAdV-26, HAdV-29, HAdV-34, HAdV-35, HAdV-37, HAdV-38, HAdV-43, HAdV-48, or HAdV-50. (**A**) Beads were washed and bound aptamers eluted by heating (5 min/95 °C). The binding efficiency was analyzed by the determination of the fluorescence intensity of elutions. Relative fluorescence intensities were normalized to HAdV-5. A detection window was found by determining the average relative fluorescence of all virus types (red dashed line) and calculating the standard deviations (dotted black lines) 75.06 ± 39.29%. Results are given as mean % of binding rel. to HAdV-5-binding + standard deviation of experiments conducted in biological triplicates (n = 3), with *p* values < 0.05 considered significant. ** denotes *p* < 0.01 and *** *p* < 0.001; ns means not significant. Detection of aptamer-binding to viral particles by dot-blot analysis. Shown is data from n = 2 biological replicates; both experiments have comparable results. (**B**) A total of 5E9 viral particles (VP) of different HAdVs, as indicated, or 3E12 VP of AAV8 were blotted on a nitrocellulose membrane and stained by Ponceau S red, demonstrating roughly equal amounts of proteins blotted on the membrane. HAdV VPs, as indicated, were specifically detected with (**C**) mouse-anti-hexon antibody (0.5 pmol) followed by rat-anti-mouse-IgG-HRP (1:20,000), (**D**) anti-HAdV-5 biotin-conjugated aptamer library (0.5 pmol), followed by streptavidin-HRP (1:5000). (**E**) An anti-Apo-RBP4 biotin-conjugated aptamer library was used as control. (**F**) Neither use of secondary antibody nor streptavidin-HRP resulted in unspecific binding. (**G**) Aptamers demonstrated specific recognition of HAdV-5 in DPBS-T and TBS-T buffered systems but not of AAV8 with + included and - without depicted components.

Given the high selectivity of aptamer library R9.1. towards HAdV-5, belonging to adenovirus species C, the potential cross-reactivity of the oligonucleotides towards other human adenovirus types was analyzed. Therefore, a total of 12 different human adenoviruses from species B (types 3, 11, 34, 35, and 50) and from species D (types 24, 26, 29, 37, 38, 43, and 48), respectively, were covalently coupled to NHS-activated magnetic beads, as described before. The harsh elution of attached vector particles and the subsequent analysis of the elution samples by reducing SDS-PAGE and the silver staining of proteins confirmed the comparable coupling efficiencies for all adenovirus types (Figure S1). Next, the Cy5-labeled aptamer library of SELEX round 9.1 was co-incubated with unloaded or Ad-particle-loaded beads. Again, beads were washed, bound aptamers were eluted, and the fluorescence intensity of elution samples was determined (Figure 5A). The binding of aptamers to HAdV-5-loaded beads was used as a reference and set to 100%. A detection window was determined by calculating the average relative fluorescence of all virus types (red dashed line) and the standard deviations (dotted black lines). Interestingly, the aptamers showed substantial cross-reactivity towards type 3, 11, 24, 26, 29, 34, 35, 38, and 43. In contrast, aptamers apparently did not bind to type 37 and less so or not at all to types 48 and 50 (Figure 5A).

As a control, the capability of the final aptamer library to bind to the adenovirus but not to adenovirus-associated virus 8 (AAV8), belonging to a different and unrelated virus family and originally discovered as a contaminant of adenoviruses, was verified in dot-blot experiments in comparison to labeling with a monoclonal mouse-anti-hexon antibody, known to recognize all adenovirus types by apparently binding to an epitope conserved across all tested adenovirus types [56] in combination with a rat-anti-mouse-IgG-HRP secondary antibody conjugate. In these experiments, the aptamers were PCR-labeled with 5’-biotin as a ligand for a streptavidin-HRP conjugate. Ponceau S red staining of the nitrocellulose membranes indicated that equal amounts of viral proteins, corresponding to a comparable number of viral particles, were used in the labeling experiments (Figure 5). As a presumably non-specific aptamer library, we chose a polyclonal library, which had previously been evolved and characterized during the development of a sensor-based assay for the quantification of the retinol-binding protein 4 (RBP4) as a potential diabetes-type-2 marker. In this case, the library was directed against apo-RBP4, the RBP4-isomer without retinol bound to the protein [46]. As expected for this “off-target” library, absolutely no detectable signal was observed for the membrane-bound viruses. Controls using only the secondary rat-anti-mouse IgG-HRP antibody and streptavidin-HRP conjugates completely failed in labeling HAdV-5 and AAV8. Also, the buffer used for the experiments had no influence on the binding capability of the aptamers (Figure 5).

**Figure 6:**
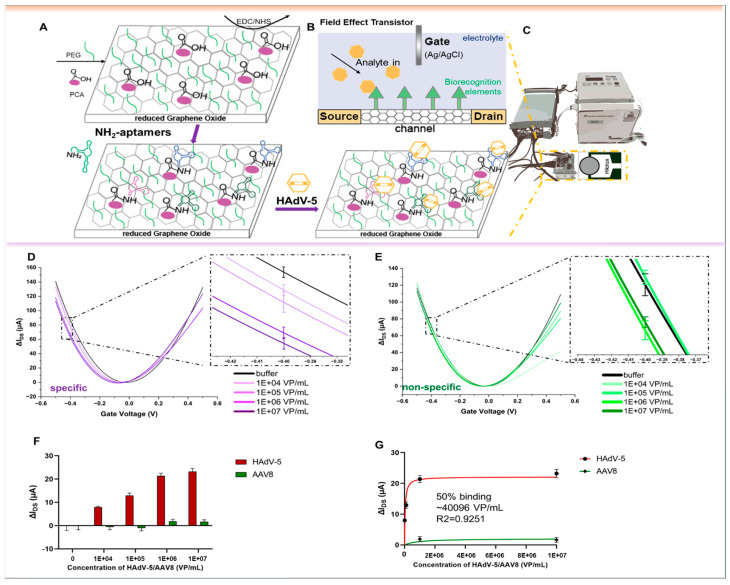
(**A**) Functionalization of rGO-FETs with polyclonal ssDNA aptamer libraries and adenovirus detection. The rGO-FET were immersed into a mixture of PyPEG (PEG pre-conjugated with pyrene) (500 µM) and 1-pyrenecarboxylic acid (PCA, 50 μM, and linker) in DMSO for 12 h at room temperature to obtain a 10:1 ratio of blocking and linking agents on the biosensor’s surface. HAdV-5 aptamer library immobilization by first activating the carboxyl groups by immersion into a solution of EDC (15 mM)/NHS (15 mM) in 150 mM PBS solution for 30 min, followed by covalent coupling of the 5′-NH_2_ modified aptamer (100 nM in milliQ grade water for 40 min at 25 °C). Specific affinity recognition of adenovirus by rGO-FET immobilized polyclonal ssDNA aptamer libraries in electrical measurements. (**B**) Typical configuration of a gFET and (**C**) devices of aptasensors. Specific rGO-FET sensing of increasing amounts (1E4 to 1E7) of (**D**) HAdV-5 viral particles by HAdV-5-R9.1 aptamer library and (**E**) AAV8 as control virus. (**F**) Recording of I_D_V_G_ curves, which are an average of a minimum of 3 individual curves with error bars showing the standard deviation. Average difference in current at −0.4 V (ΔI_DS_) upon addition of increasing concentrations of viral particles and (**G**) resulting nonlinear curve fitting according to the one site-specific binding model in GraphPad Prism 8, with the 50% binding around 40,096 VP/mL, whereas AAV8 did not show a concentration-specific correlation with very low signals (green). All experiments were conducted in triplicate with error bars representing standard deviations.

To evaluate the performance of the aptamer libraries for biosensing, they were used as biorecognition elements on an electrolyte-gated reduced Graphene Oxide Field-Effect Transistor (EG-rGO-FET). The rGO-FET was fabricated following the procedure outlined in previous studies [46,57,58]. Measurements were conducted in a Micrux all-in-one flow cell with an Ag/AgCl electrode serving as the gate. Special aptamer libraries were created with primers that included an amine functional group at the 5’-end. This aptamer library was then immobilized on a pyrene carboxylic acid-functionalized reduced Graphene Oxide surface through EDC–NHS coupling, following previously published procedures (see Figure 6A) [46,57,58]. The binding of analytes impacts the charge transfer properties of the device, which can be detected through ‘I_DS_V_G_-curves’. In these curves, the gate voltage (V_G_) is varied between −0.5 and 0.5 V, while the source–drain current (I_DS_) is measured. Higher voltages were avoided to prevent damage to biological components. Shifts in the I_DS_V_G_ characteristics are attributed to variations in charge carrier mobility, which arise from changes in charge distribution at the channel–electrolyte interface [43] (Figure 6B,C). This distribution is altered during analyte binding events, such as with charged analytes like proteins, or due to structural changes in the negatively charged aptamer backbone upon analyte binding. Analyte binding can be monitored in real time by tracking I_DS_ at a constant gate potential during analyte addition [59,60,61] or by recording I_DS_V_G_ curves after the analyte is introduced. In the real experiments, increasing numbers of virus particles HAdV-5 and AAV8, as the control virus, were measured on the rGO-FET chips, and the instruments’ response signals were recorded. Prior to the experiments, the surface functionalization of the individual respective chips was also recorded to verify the deposition of aptamers on the surface (“layer-by-layer” functionalization) in the same experimental set up (Figure S2). The response signals increased specifically with the number of HAdV-5 (Figure 6D,F), delivering a nonlinear fit according to the one site-specific binding model (Figure 6G), whereas AAV8 did not show a concentration-specific correlation, although, at high concentrations, it did show unspecific (“noise”) signals in low dilutions (Figure 6E,F). As a consequence, no stable fit could be obtained with the same model (Figure 6G). Because the slope and capacitive effects can differ across branches, we quantified the response in the steep segment of the hole branch (left side from the Dirac point) and additionally analyzed the bias-independent Dirac point shifts with both metrics, yielding consistent trends upon target binding (Figure S3).

## 4. Discussion

The combination of aptamers as specificity-determining entities and functionalized reduced graphene transistors has entered the stage of possible real-life applications, for example, as the technological basis for the development of point-of-care devices [62,63]. This, in turn, may also suggest the readiness for applications in (bio)technology, e.g., in future on-line or at-line monitoring techniques in production processes for cells, proteins, or therapeutic viruses. The crucial process in the developmental workflow towards a functional aptamer-based rGO-FET for the specific quantification of a specific target is the evolutionary selection process against this molecule, cell, tissue, or virus particle. The SELEX against HAdV-5 succeeded in only nine selection rounds, with two final polishing rounds only consisting of a counter-selection step without the target involved. This focused library allowed for preliminary fluorescence-based testing against magnetic beads carrying virus particles, as well as a dot-blot procedure with specific discrimination of the target virus from AAV8 used as a control derived from a different virus family. It was easily possible to adapt conventional detection methods for dot-blot or Western-blot analyses, based on conjugates with horse radish peroxidase and streptavidin, to use it with the aptamer library simply by modifying it with a biotin label at the 5’-end. Adenoviruses are non-enveloped viruses (i.e., not containing a lipid membrane), containing a capsid with mainly three proteins that are surface/solvent exposed: Hexon (720 monomers, organized as 240 trimers), Penton base (60 monomers organized as 12 pentamers), and Fiber (36 monomers organized as 12 trimers) [64]. Thus, the isolated aptamers will mainly be directed against these three proteins. Since the library is polyclonal, it can be expected that the library will recognize all three proteins of HAdV-5. Depending on the evolutionary conservation of adenovirus, one would expect that some of the aptamers might recognize epitopes that are common to different adenovirus types, and some epitopes might be more specific to HAdV-5. In fact, our data showed that the polyclonal aptamer library selected cross reactions with a large collection of adenovirus types belonging to different adenovirus species. This indicated that at least some of the selected aptamers contained in the polyclonal library recognized one or several epitopes that are common to the different adenovirus types. We currently cannot explain the lack of detection of HAdV-37 by the HAdV-5-derived and Cy5-labeled aptamers, while the same adenovirus type was readily detected upon a dot-blot analysis. One explanation could be that the cross-linking of HAdV-37 to the magnetic beads may have shielded an epitope that is solvent-exposed/accessible both in HAdV-5 and in HAdV-37 when the viruses are blotted onto the membrane under non-denaturing conditions, while the epitope is not accessible in HAdV-37 when the virus is linked to the bi-functional coupling reagent and/or linked to the magnetic beads. In the future, it will be interesting to test individual aptamers derived from the library for specificity against different adenovirus types. While aptamers have been tested as targeting ligands to modify the natural tropism of viruses, including adenoviruses [65,66], applications of aptamers for sensing adenoviruses appear to be rather limited. Adenovirus-specific aptamers have been used successfully in a surface-enhanced Raman spectroscopy-based concept [67] and in electronic measurements using aptamers in combination with a solid-state nanopore system, reaching a detection limit of 1E4 viral particles [68]. The aim of this study—to show the general suitability of SELEX-derived focused aptamer libraries as binding entities on gFETs that are also for quantification of adenoviruses—was achieved by demonstrating not only an appropriate specificity allowing for the discrimination of HAdV-5 from AAV8 but also by reaching a reasonably high sensitivity of 1E4 VP/mL without any further optimization. Upon increasing the HAdV-5 concentration, we consistently observed a leftward shift in the Dirac point. Since both the adenovirus and the nucleic acid aptamers are negatively charged under physiological conditions, a rightward shift might be expected from a simple charge-addition model. However, similar leftward shifts have been reported in other nucleic acid FET sensors, where ΔV_Dirac_ was attributed to doping effects from DNA/viral RNA hybridization [53,69]. In our system, the large size of the virus relative to the short Debye screening length in 1 × PBS makes it unlikely that the capsid charge is directly detected. Instead, the electrical response likely reflects the conformational or orientational rearrangements of the aptamer layer upon virus binding, which effectively redistributes the negative charge within the sensing range of the channel. Similar observations have been reported for FET sensors detecting small or neutral molecules (e.g., serotonin, glucose), where signal transduction occurs via charge rearrangement of the aptamer receptor on the channel, effectively overcoming Debye-length limitations [70,71,72,73]. The Debye screening length at the graphene–electrolyte interface depends strongly on ionic strength, and for a 2D charge distribution, 1 × PBS corresponds to ~0.7 nm, whereas diluting to 0.001 × PBS increases the Debye length to ~23 nm. In this study, physiological 1 × PBS was used to reflect realistic sample conditions, meaning that only charges very close to the rGO surface, such as those from the aptamer layer, contribute significantly to the FET response [74]. The observed responses were more pronounced in the hole accumulation branch (left of the Dirac point), consistent with the predominantly p-type behavior of rGO under electrolyte gating, i.e., transport is dominated by holes introduced by environmental doping. Changes in local surface potential caused by an aptamer–virus interaction are transduced more efficiently, whereas the electron branch is less sensitive and more affected by capacitive contributions; hence, the analytical signal was analyzed from this branch and complemented by the observed Dirac point shifts as a bias-independent metric. Our sensor is still based on a focused aptamer library, which can serve as the resource to isolate individual aptamers by bioinformatic analyses and their subsequent chemical synthesis, in-depth characterization, and later performance evaluation, also on our rGO-FET sensors. A tenfold improvement in sensitivity can be expected due to the drastic increase in surface loading density with exclusively specific aptamers, as shown by a similar aptasensor system for the quantification of a diabetes marker protein [75]. Typical numbers in a disease range from 10^9^ to 10^12^ virus particles per milliliter (e.g., in respiratory fluids) [76,77] and also up to 10^12^–10^13^ particles in cell culture media, as delivered by the biotechnological production of adenoviruses [78]. Hence, if the employment of the aptamer-based sensing system is desired in the quantification of virus particles in human body fluids for diagnostic purposes or their detection in cell culture supernatants after virus production for manufacturing viral vectors for gene therapy, the sensitivity obtained provides a 5–8 order of magnitude margin for dilution to circumvent the technical fluid-based limitations, such as with increased viscosity (e.g., respiratory fluids) and/or high concentrations of contaminating proteins (e.g., in culture broth). Current detection methods for adenoviruses rely mainly on PCR-based assays, lateral flow tests, and ELISA [79]. PCR-based approaches, including RT-PCR, remain the clinical gold standard due to their high sensitivity (down to ~100 VP/mL) and specificity, but they require DNA extraction, multiple amplification steps, and specialized laboratory infrastructure, leading to turnaround times of at least 1–2 h [80]. Colloidal gold-based lateral flow assays exist, which are already commercially available, with a number of groups working on their improvement concerning sensitivity, specificity, and easy operation [81]. Relevant respective commercial kits (assays) (EZER™ ADV Antigen Rapid Test. Available online at: https://genesis-ivd.com/index/respiratory-pathogen-rapid-test/13.html (accessed on 28 August 2025)) [70] have been described to have higher detection limits of 1E3 to 1E4 VP/mL, and specificity can be limited. ELISA is generally applied for the indirect detection of the host immune response rather than the virus itself; for example, anti-adenovirus IgA, IgG, and IgM ELISA kits only detect antibodies in plasma or serum [82,83], making such methods unsuitable for the rapid identification of acute viral infections. The aptamer-rGO-FET biosensor presented here enables the label-free and fast detection of intact adenovirus particles directly in the physiological buffer, with measurements completed within minutes rather than hours. The use of aptamers as recognition elements offers further advantages over antibodies, including higher stability, lower production costs, and the possibility of addressing specificity and sensitivity with the selection of individual aptamers. While full clinical benchmarking against PCR and commercial test kits is beyond the scope of the present study, our results demonstrate the potential for aptamer-rGO-FETs as rapid, inexpensive, and portable diagnostic platforms that could complement existing methods and build the basis for future point-of-care applications. The polyclonal or focused library presented in this study has shown its principal appropriateness for sensitive and specific quantification of the target virus. Such characterized libraries typically offer the opportunity to deliver selected and probably better performing individual aptamers after comparative next-generation sequencing, extensive bioinformatic analyses, chemical synthesis of the candidate aptamers, and their subsequent in-depth characterization for suitability in the desired experimental environment. Even in the case of an undoubtedly achievable gain in sensitivity by the use of individual aptamers, it appears to not be mandatory in a given application; their supply by chemical synthesis may be advantageous on the way to a marketable product, since this technology is easily scalable and highly reproducible. The use of polyclonal libraries as the aptamer functionality in technical devices or diagnostic techniques requires a (PCR-based) production technology to synthesize the respective mixture of productive individual aptamer sequences (i.e., the complete sequence space of the functional library). This has been shown to be possible with high reproducibility over generations of library production processes and thus different batches of libraries perfectly maintaining their specificity [17]. Moreover, in the case of aptamers intended to specifically detect the opportunistic human pathogenic bacterium *Pseudomonas aeruginosa*, the polyclonal or focused library have been shown to outperform individual aptamers in the precise and robust detection of a broad target spectrum (e.g., different clinical isolates of the same pathogenic bacterial species) [17]. With the results on the development of the anti-HAdV-5 aptamer library, their characterization, and the demonstration of its suitability for the construction of a functional gFET sensor, we prove that the evolution of focused polyclonal aptamer libraries and their use in sensors instead of single aptamers can serve as a successful strategy. A follow-up study will take advantage of these preliminary but important results by isolating individual aptamers upon bioinformatic analyses and their in-depth characterization with the complete set of adenoviruses on gFET sensors and will evaluate not only strain specificity but also identify the molecular targets. Moreover, we hope to inspire scientists and product developers to make use of this strategy either alone, for the development of assay systems, or in combination with electronic or optical principles for the construction of next-generation sensing technologies towards improved diagnostics and/or process monitoring in biotechnology.

## Figures and Tables

**Table 1:** Selection conditions of anti- HAdV-5 SELEX. Rounds of SELEX, applied amount of aptamer library, counter-SELEX and target-SELEX steps, washing regimes, and the amount of BSA/tRNA for blocking.

SELEX Round	Aptamer [pmol]	Counter SELEX	Target SELEX	BSA/tRNA [pmol]	Washing Steps
1	100	1	1	600	3 × HEPES
2	10	1	1	900	3 × HEPES
3	10	1	1	1200	3 × HEPES
4	10	1	1	1500	3 × HEPES
5	1	1	1	1800	3 × HEPES + 0.1% BSA
6	1	1	1	2100	4 × HEPES + 0.1% BSA
7	1	1	1	2400	5 × HEPES + 0.1% BSA
8	1	1	1	2700	6 × HEPES + 0.1% BSA
9	0.1	1	1	3000	7 × HEPES + 0.1% BSA
9.1	0.1	2	/	/	/
9.2	0.01	2	/	/	/

## Data Availability

The original contributions presented in this study are included in the article/supplementary material. Further inquiries can be directed to the corresponding author(s).

## Supplementary Materials

The following supporting information can be downloaded at: https://www.mdpi.com/article/10.3390/bios15090605/s1, Figure S1. Covalent coupling of adenoviruses to NHS-activated beads. 1E9 VP/µL bead in a total volume of 20 µL/µL bead of the indicated vector or virus type were covalently attached to NHS-activated magnetic beads. Elution was done by addition of SDS-loading buffer and heating for 15 min at 96 °C. Eluted proteins were separated under reducing conditions by SDS-PAGE and comparable coupling efficiency for all types was confirmed by visualization of viral proteins by silver staining.; Figure S2. Layer-by-layer functionalization of reduced graphene chip surfaces using PyPEG linkers and subsequently EDC/NHS-mediated coupling of NH_2_-labeled anti-HAdV-5 aptamer library for each chip used for the experiments shown in Figure 6. Figure S3. Dirac point shifts (∆_Dirac)_ resulting from I_DS_V_G_ transfer curves measured in Figure 6. Error bars represent standard deviations.

## References

[1] Ellington A.D., Szostak J.W. (1990). In Vitro Selection of RNA Molecules That Bind Specific Ligands. Nature.

[2] Tuerk C., Gold L. (1990). Systematic Evolution of Ligands by Exponential Enrichment: RNA Ligands to Bacteriophage T4 DNA Polymerase. Science.

[3] Ku T.-H., Zhang T., Luo H., Yen T.M., Chen P.-W., Han Y., Lo Y.-H. (2015). Nucleic Acid Aptamers: An Emerging Tool for Biotechnology and Biomedical Sensing. Sensors.

[4] Ilgu M., Nilsen-Hamilton M. (2016). Aptamers in Analytics. Analyst.

[5] Bunka D.H.J., Stockley P.G. (2006). Aptamers Come of Age—At Last. Nat. Rev. Microbiol..

[6] Chopra A., Shukla R., Sharma T.K. (2014). Aptamers as an Emerging Player in Biology. Front. Genet..

[7] Chen A., Yang S. (2015). Replacing Antibodies with Aptamers in Lateral Flow Immunoassay. Biosens. Bioelectron..

[8] Guo W., Zhang C., Ma T., Liu X., Chen Z., Li S., Deng Y. (2021). Advances in Aptamer Screening and Aptasensors’ Detection of Heavy Metal Ions. J. Nanobiotechnol..

[9] Kusumawati A., Mustopa A.Z., Wibawan I.W.T., Setiyono A., Sudarwanto M.B. (2022). A Sequential Toggle Cell-SELEX DNA Aptamer for Targeting Staphylococcus Aureus, Streptococcus Agalactiae, and Escherichia Coli Bacteria. J. Genet. Eng. Biotechnol..

[10] Li L., Wan J., Wen X., Guo Q., Jiang H., Wang J., Ren Y., Wang K. (2021). Identification of a New DNA Aptamer by Tissue-SELEX for Cancer Recognition and Imaging. Anal. Chem..

[11] Murakami K., Izuo N., Bitan G. (2022). Aptamers Targeting Amyloidogenic Proteins and Their Emerging Role in Neurodegenerative Diseases. J. Biol. Chem..

[12] Sefah K., Shangguan D., Xiong X., O’Donoghue M.B., Tan W. (2010). Development of DNA Aptamers Using Cell-SELEX. Nat. Protoc..

[13] Yang J., Bowser M.T. (2013). Capillary Electrophoresis-SELEX Selection of Catalytic DNA Aptamers for a Small-Molecule Porphyrin Target. Anal. Chem..

[14] Zhong W., Pu Y., Tan W., Liu J., Liao J., Liu B., Chen K., Yu B., Hu Y., Deng Y. (2019). Identification and Application of an Aptamer Targeting Papillary Thyroid Carcinoma Using Tissue-SELEX. Anal. Chem..

[15] Tan S.Y., Acquah C., Sidhu A., Ongkudon C.M., Yon L.S., Danquah M.K. (2016). SELEX Modifications and Bioanalytical Techniques for Aptamer–Target Binding Characterization. Crit. Rev. Anal. Chem..

[16] Stoltenburg R., Reinemann C., Strehlitz B. (2005). FluMag-SELEX as an Advantageous Method for DNA Aptamer Selection. Anal. Bioanal. Chem..

[17] Kubiczek D., Raber H., Bodenberger N., Oswald T., Sahan M., Mayer D., Wiese S., Stenger S., Weil T., Rosenau F. (2020). The Diversity of a Polyclonal FluCell-SELEX Library Outperforms Individual Aptamers as Emerging Diagnostic Tools for the Identification of Carbapenem Resistant Pseudomonas Aeruginosa. Chemistry.

[18] Raber H.F., Kubiczek D.H., Bodenberger N., Kissmann A.K., D’souza D., Hu X., Mayer D., Xu P., Knippschild U., Spellerberg B. (2021). FluCell-SELEX Aptamers as Specific Binding Molecules for Diagnostics of the Health Relevant Gut Bacterium Akkermansia Muciniphila. Int. J. Mol. Sci..

[19] Xing H., Zhang Y., Krämer M., Kissmann A.K., Henkel M., Weil T., Knippschild U., Rosenau F. (2022). A Polyclonal Selex Aptamer Library Directly Allows Specific Labelling of the Human Gut Bacterium Blautia Producta without Isolating Individual Aptamers. Molecules.

[20] Xing H., Kissmann A.K., Raber H.F., Krämer M., Amann V., Kohn K., Weil T., Rosenau F. (2021). Polyclonal Aptamers for Specific Fluorescence Labeling and Quantification of the Health Relevant Human Gut Bacterium Parabacteroides Distasonis. Microorganisms.

[21] Zhang Y., Xing H., Bolotnikov G., Krämer M., Gotzmann N., Knippschild U., Kissmann A.K., Rosenau F. (2023). Enriched Aptamer Libraries in Fluorescence-Based Assays for Rikenella Microfusus-Specific Gut Microbiome Analyses. Microorganisms.

[22] Xing H., Zhang Y., Krämer M., Kissmann A.K., Amann V., Raber H.F., Weil T., Stieger K.R., Knippschild U., Henkel M. (2022). A Polyclonal Aptamer Library for the Specific Binding of the Gut Bacterium Roseburia Intestinalis in Mixtures with Other Gut Microbiome Bacteria and Human Stool Samples. Int. J. Mol. Sci..

[23] Kneißle K., Krämer M., Kissmann A.K., Xing H., Müller F., Amann V., Noschka R., Gottschalk K.E., Bozdogan A., Andersson J. (2022). A Polyclonal SELEX Aptamer Library Allows Differentiation of *Candida albicans*, *C. auris* and *C. parapsilosis* Cells from Human Dermal Fibroblasts. J. Fungi.

[24] Zhang Y., Xing H., Bolotnikov G., Krämer M., Bozdogan A., Kissmann A.K., Weil T., Spellerberg B., Stenger S., Rosenau F. (2024). Robust Fluorometric Aptamer Assay for Direct and Rapid Detection of Clinical Isolates of Candida Spec. Int. J. Mol. Sci..

[25] Kissmann A.K., Wolf D., Krämer M., Müller F., Amann V., Xing H., Gottschalk K.E., Weil T., Eichmann R., Schäfer P. (2022). Polyclonal Aptamer Libraries from a FluRoot-SELEX for the Specific Labeling of the Apical and Elongation/Differentiation Zones of Arabidopsis Thaliana Roots. Int. J. Mol. Sci..

[26] Alemany R. (2014). Oncolytic Adenoviruses in Cancer Treatment. Biomedicines.

[27] Farrera-Sal M., Moya-Borrego L., Bazan-Peregrino M., Alemany R. (2021). Evolving Status of Clinical Immunotherapy with Oncolytic Adenovirus. Clin. Cancer Res..

[28] Suleman S., Schrubaji K., Filippou C., Ignatova S., Hewitson P., Huddleston J., Karda R., Waddington S.N., Themis M. (2022). Rapid and Inexpensive Purification of Adenovirus Vectors Using an Optimised Aqueous Two-Phase Technology. J. Virol. Methods.

[29] Dicks M.D.J., Spencer A.J., Edwards N.J., Wadell G., Bojang K., Gilbert S.C., Hill A.V.S., Cottingham M.G. (2012). A Novel Chimpanzee Adenovirus Vector with Low Human Seroprevalence: Improved Systems for Vector Derivation and Comparative Immunogenicity. PLoS ONE.

[30] Bos R., Rutten L., van der Lubbe J.E.M., Bakkers M.J.G., Hardenberg G., Wegmann F., Zuijdgeest D., de Wilde A.H., Koornneef A., Verwilligen A. (2020). Ad26 Vector-Based COVID-19 Vaccine Encoding a Prefusion-Stabilized SARS-CoV-2 Spike Immunogen Induces Potent Humoral and Cellular Immune Responses. NPJ Vaccines.

[31] Wu S., Zhong G., Zhang J., Shuai L., Zhang Z., Wen Z., Wang B., Zhao Z., Song X., Chen Y. (2020). A Single Dose of an Adenovirus-Vectored Vaccine Provides Protection against SARS-CoV-2 Challenge. Nat. Commun..

[32] Pilely K., Johansen M.R., Lund R.R., Kofoed T., Jørgensen T.K., Skriver L., Mørtz E. (2021). Monitoring Process-Related Impurities in Biologics–Host Cell Protein Analysis. Anal. Bioanal. Chem..

[33] Madisch I., Wölfel R., Harste G., Pommer H., Heim A. (2006). Molecular Identification of Adenovirus Sequences: A Rapid Scheme for Early Typing of Human Adenoviruses in Diagnostic Samples of Immunocompetent and Immunodeficient Patients. J. Med. Virol..

[34] Heim A., Ebnet C., Harste G., Pring-Åkerblom P. (2003). Rapid and Quantitative Detection of Human Adenovirus DNA by Real-Time PCR. J. Med. Virol.

[35] Ban D.K., Hajian R., Chan M., Abdolrahimi S., Barron F., Datta S., Aran K. (2024). Real-Time Monitoring in Biomanufacturing with Graphene Field-Effect Transistor Sensors: Detection of PH, Glucose, and Antibodies. GEN Biotechnol..

[36] Saiki P.Y., Rufino F.C., de Almeida C.R., Sales G.M., de Oliveira A.N., Larrude D.R.G., Teixeira R.C., Diniz J.A., Catharino R.R. (2025). Sensor Detection of Shelf Life: A Multi-Technique Food Analytical Platform for Mushroom Analysis. Food Res. Int..

[37] Kaiser D., Meyerbroeker N., Purschke W., Sell S., Neumann C., Winter A., Tang Z., Hüger D., Maasch C., Bethge L. (2024). Ultrasensitive Detection of Chemokines in Clinical Samples with Graphene-Based Field-Effect Transistors. Adv. Mater..

[38] Geiwitz M., Page O.R., Marello T., Nichols M.E., Kumar N., Hummel S., Belosevich V., Ma Q., van Opijnen T., Batten B. (2024). Graphene Multiplexed Sensor for Point-of-Need Viral Wastewater-Based Epidemiology. ACS Appl. Bio Mater..

[39] Fomin M., Jorde L., Steinbach F., You C., Meyer C. (2023). Liquid-Gated Graphene Field-Effect Transistors for Biosensing on Lipid Monolayers. Phys. Status Solidi B Basic Res..

[40] Selvarajan R.S., Rahim R.A., Majlis B.Y., Gopinath S.C.B., Hamzah A.A. (2020). Ultrasensitive and Highly Selective Graphene-Based Field-Effect Transistor Biosensor for Anti-Diuretic Hormone Detection. Sensors.

[41] Danielson E., Sontakke V.A., Porkovich A.J., Wang Z., Kumar P., Ziadi Z., Yokobayashi Y., Sowwan M. (2020). Graphene Based Field-Effect Transistor Biosensors Functionalized Using Gas-Phase Synthesized Gold Nanoparticles. Sens. Actuators B Chem..

[42] Béraud A., Sauvage M., Bazán C.M., Tie M., Bencherif A., Bouilly D. (2021). Graphene Field-Effect Transistors as Bioanalytical Sensors: Design, Operation and Performance. Analyst.

[43] Reiner-Rozman C., Larisika M., Nowak C., Knoll W. (2015). Graphene-Based Liquid-Gated Field Effect Transistor for Biosensing: Theory and Experiments. Biosens. Bioelectron..

[44] Wang G., Zhang M., Zhu M., Zhang T., Qian X., Liu Y., Ma X., Dai C., Wei D., Zhu Z. (2025). Ultraprecise Detection of Influenza Virus by Antibody-Modified Graphene Transistors. Sensors.

[45] Luo Y., Zhu B., Zhu C., Lai P., Taylor J., Honney C., Nutsford A., Ma C., Chen H., Aw K.C. (2025). Ultrasensitive, Real-Time Detection of Viral Antigens and RNA Enabled by Scalable Graphene-Based FET Sensors for Pathogen Detection: A Case Study on COVID-19. ACS Sens..

[46] Kissmann A.K., Andersson J., Bozdogan A., Amann V., Krämer M., Xing H., Raber H.F., Kubiczek D.H., Aspermair P., Knoll W. (2022). Polyclonal Aptamer Libraries as Binding Entities on a Graphene FET Based Biosensor for the Discrimination of Apo- and Holo-Retinol Binding Protein 4. Nanoscale Horiz..

[47] Ono T., Kanai Y., Inoue K., Watanabe Y., Nakakita S.I., Kawahara T., Suzuki Y., Matsumoto K. (2019). Electrical Biosensing at Physiological Ionic Strength Using Graphene Field-Effect Transistor in Femtoliter Microdroplet. Nano Lett..

[48] Wang S., Hossain M.Z., Shinozuka K., Shimizu N., Kitada S., Suzuki T., Ichige R., Kuwana A., Kobayashi H. (2020). Graphene Field-Effect Transistor Biosensor for Detection of Biotin with Ultrahigh Sensitivity and Specificity. Biosens. Bioelectron..

[49] Vu C.-A., Chen W.-Y. (2020). Predicting Future Prospects of Aptamers in Field-Effect Transistor Biosensors. Molecules.

[50] Shaban S.M., Kim D.-H. (2021). Recent Advances in Aptamer Sensors. Sensors.

[51] Sánchez-Tirado E., Agüí L., González-Cortés A., Campuzano S., Yáñez-Sedeño P., Pingarrón J.M. (2023). Electrochemical (Bio)Sensing Devices for Human-Microbiome-Related Biomarkers. Sensors.

[52] Kissmann A.K., Bolotnikov G., Li R., Müller F., Xing H., Krämer M., Gottschalk K.E., Andersson J., Weil T., Rosenau F. (2024). IMPATIENT-QPCR: Monitoring SELEX Success during in Vitro Aptamer Evolution. Appl. Microbiol. Biotechnol..

[53] Herdina A.N., Bozdogan A., Aspermair P., Dostalek J., Klausberger M., Lingg N., Cserjan-Puschmann M., Aguilar P.P., Auer S., Demirtas H. (2025). Bridging Basic Science and Applied Diagnostics: Comprehensive Viral Diagnostics Enabled by Graphene-Based Electronic Biosensor Technology Advancements. Biosens. Bioelectron..

[54] Reiner-Rozman C., Hasler R., Andersson J., Rodrigues T., Bozdogan A., Bintinger J., Aspermair P. (2021). The Top Performer: Towards Optimized Parameters for Reduced Graphene Oxide Uniformity by Spin Coating. Micro Nano Lett..

[55] Leikas A.J., Ylä-Herttuala S., Hartikainen J.E.K. (2023). Adenoviral Gene Therapy Vectors in Clinical Use—Basic Aspects with a Special Reference to Replication-Competent Adenovirus Formation and Its Impact on Clinical Safety. Int. J. Mol. Sci..

[56] Michalik S., Siegerist F., Palankar R., Franzke K., Schindler M., Reder A., Seifert U., Cammann C., Wesche J., Steil L. (2022). Comparative Analysis of ChAdOx1 NCoV-19 and Ad26.COV2.S SARS-CoV-2 Vector Vaccines. Haematologica.

[57] Xing H., Zhang Y., Li R., Ruzicka H.M., Hain C., Andersson J., Bozdogan A., Henkel M., Knippschild U., Hasler R. (2024). A Blautia Producta Specific GFET-Based Aptasensor for Quantitative Monitoring of Microbiome Quality. Nanoscale Horiz..

[58] Zhang Y., Xing H., Li R., Andersson J., Bozdogan A., Strassl R., Draphoen B., Lindén M., Henkel M., Knippschild U. (2025). Specific GFET-Based Aptasensors for Monitoring of Microbiome Quality: Quantification of the Enteric Health-Relevant Bacterium Roseburia Intestinalis. Adv. Heal. Mater..

[59] Li K., Tu J., Zhang Y., Jin D., Li T., Li J., Ni W., Xiao M.M., Zhang Z.Y., Zhang G.J. (2022). Ultrasensitive Detection of Exosomal MiRNA with PMO-Graphene Quantum Dots-Functionalized Field-Effect Transistor Biosensor. iScience.

[60] Thakur B., Zhou G., Chang J., Pu H., Jin B., Sui X., Yuan X., Yang C.H., Magruder M., Chen J. (2018). Rapid Detection of Single E. Coli Bacteria Using a Graphene-Based Field-Effect Transistor Device. Biosens. Bioelectron..

[61] Zhou L., Mao H., Wu C., Tang L., Wu Z., Sun H., Zhang H., Zhou H., Jia C., Jin Q. (2017). Label-Free Graphene Biosensor Targeting Cancer Molecules Based on Non-Covalent Modification. Biosens. Bioelectron..

[62] Thriveni G., Ghosh K. (2022). Advancement and Challenges of Biosensing Using Field Effect Transistors. Biosensors.

[63] Forsyth R., Devadoss A., Guy O. (2017). Graphene Field Effect Transistors for Biomedical Applications: Current Status and Future Prospects. Diagnostics.

[64] Gallardo J., Pérez-Illana M., Martín-González N., San Martín C. (2021). Adenovirus Structure: What Is New?. Int. J. Mol. Sci..

[65] Chen H., Zheng X., Di B.Y., Wang D., Zhang Y., Xia H., Mao Q. (2013). Aptamer Modification Improves the Adenoviral Transduction of Malignant Glioma Cells. J. Biotechnol..

[66] Liu Z., Sun X., Xiao S., Lin Y., Li C., Hao N., Zhou M., Deng R., Ke S., Zhong Z. (2017). Characterization of Aptamer-Mediated Gene Delivery System for Liver Cancer Therapy. Oncotarget.

[67] Kukushkin V., Ambartsumyan O., Subekin A., Astrakhantseva A., Gushchin V., Nikonova A., Dorofeeva A., Zverev V., Keshek A., Meshcheryakova N. (2023). Multiplex Lithographic SERS Aptasensor for Detection of Several Respiratory Viruses in One Pot. Int. J. Mol. Sci..

[68] Peinetti A.S., Lake R.J., Cong W., Cooper L., Wu Y., Ma Y., Pawel G.T., Toimil-Molares M.E., Trautmann C., Rong L. (2021). Direct Detection of Human Adenovirus or SARS-CoV-2 with Ability to Inform Infectivity Using DNA Aptamer-Nanopore Sensors. Sci. Adv..

[69] Szunerits S., Rodrigues T., Bagale R., Happy H., Boukherroub R., Knoll W. (2024). Graphene-Based Field-Effect Transistors for Biosensing: Where Is the Field Heading To?. Anal. Bioanal. Chem..

[70] Nakatsuka N., Yang K.-A., Abendroth J.M., Cheung K.M., Xu X., Yang H., Zhao C., Zhu B., Rim Y.S., Yang Y. (2018). Aptamer–Field-Effect Transistors Overcome Debye Length Limitations for Small-Molecule Sensing. Science.

[71] Sekhon S., Bayford R., Demosthenous A. (2025). Capacitive Sensors for Label-Free Detection in High-Ionic-Strength Bodily Fluids: A Review. Biosensors.

[72] Hu W.-P., Wang J.-S., Chiu Y.-P., Huang T.-C., Yadlapalli B.K., Chen W.-Y. (2025). The Optimal Ionic Concentration of Sensing Buffer in the Detection of RNA by Using the DNA Probe with the Silicon Nanowire Field-Effect Transistor (SiNW-FET). Talanta.

[73] Wang L., Bao L., Qiao L., Wang J., Wang Y., Fu W., Zhang X. (2025). Epitope-Imprinted Field-Effect Transistors Overcome Debye Length Limitations for Label-Free Protein Detection. Nano Lett..

[74] Rodrigues T., Mishyn V., Leroux Y.R., Butruille L., Woitrain E., Barras A., Aspermair P., Happy H., Kleber C., Boukherroub R. (2022). Highly Performing Graphene-Based Field Effect Transistor for the Differentiation between Mild-Moderate-Severe Myocardial Injury. Nano Today.

[75] Xing H., Li R., Zhang Y., Rajpal S., Bolotnikov G., Gruber D., Ruzicka H.-M., Andersson J., Bozdogan A., Herdina A.N. (2025). Single-Sequence Based GFET-Aptasensors for the Discrimination of Apo–and Holo-RBP4 in Human Serum. https://ssrn.com/abstract=5345344.

[76] Schilham M.W., Claas E.C., Van Zaane W., Heemskerk B., Vossen J.M., Lankester A.C., Toes R.E., Echavarria M., Kroes A.C., Van Tol M.J. (2002). High Levels of Adenovirus DNA in Serum Correlate with Fatal Outcome of Adenovirus Infection in Children after Allogeneic Stem-Cell Transplantation. Clin. Infect. Dis..

[77] Subramanian T., Vijayalingam S., Chinnadurai G. (2006). Genetic Identification of Adenovirus Type 5 Genes That Influence Viral Spread. J. Virol..

[78] Cashdollar J.L., Huff E., Ryu H., Grimm A.C. (2016). The Influence of Incubation Time on Adenovirus Quantitation in A549 Cells by Most Probable Number. J. Virol. Methods.

[79] Li X., Zhao C., Hou G., Sun Z., Liu X., Ding Y., Fang Y., Liu Q. (2025). Simultaneously Ultrasensitive and Differential Detection of SARS-CoV-2, Adenovirus and Influenza a Virus Using Multiplex Fluorescence Lateral Flow Immunoassay. Front. Immunol..

[80] Zhao H., Yang Y., Lyu J., Ren X., Cheng W. (2021). Development and Application of a Method to Detect 27 Respiratory Pathogens Using Multiplex RT-PCR Combined with MassARRAY Technology. BMC Infect. Dis..

[81] EZERTM ADV Antigen Rapid Test. https://genesis-ivd.com/index/respiratory-pathogen-rapid-test/13.html.

[82] Roggendorf M., Wigand R., Deinhardt F., Frösner G.G. (1982). Enzyme-Linked Immunosorbent Assay for Acute Adenovirus Infection. J. Virol. Methods.

[83] Zhang Y., Qian L., Chen K., Gu S., Wang J., Meng Z., Li Y., Wang P. (2022). Intraperitoneal Oncolytic Virotherapy for Patients with Malignant Ascites: Characterization of Clinical Efficacy and Antitumor Immune Response. Mol. Ther. Oncolytics.

