# Supplementary material for: An Aptamer-Based gFET-Sensor for Specific Quantification of Gene Therapeutic Human Adenovirus Type 5
Source: Biosensors (Basel). 2025 Sep 14;15(9):605. doi: 10.3390/bios15090605 (PMC12467633; doi:10.3390/bios15090605)
Supplement: Supplementary file 1 [file biosensors-15-00605-s001.zip › biosensors-3776428-supplementary.pdf]

Supplementary Information

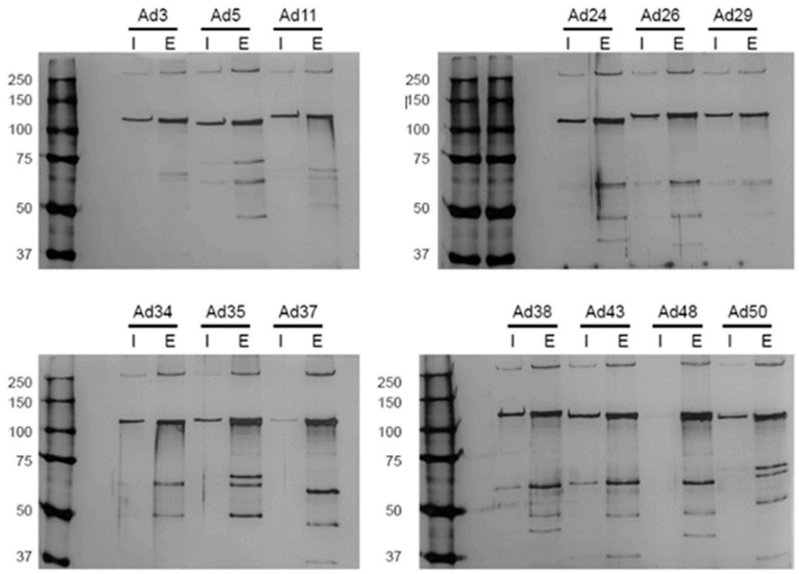

Figure S1. Covalent coupling of adenoviruses to NHS-activated beads. 1E9 VP/µL bead in a total volume of 20 µL/µL bead of the indicated vector or virus type were covalently attached to NHS-activated magnetic beads. Elution was done by addition of SDS-loading buffer and heating for 15 min at 96 °C. Eluted proteins were separated under reducing conditions by SDS-PAGE and comparable coupling efficiency for all types was confirmed by visualization of viral proteins by silver staining.

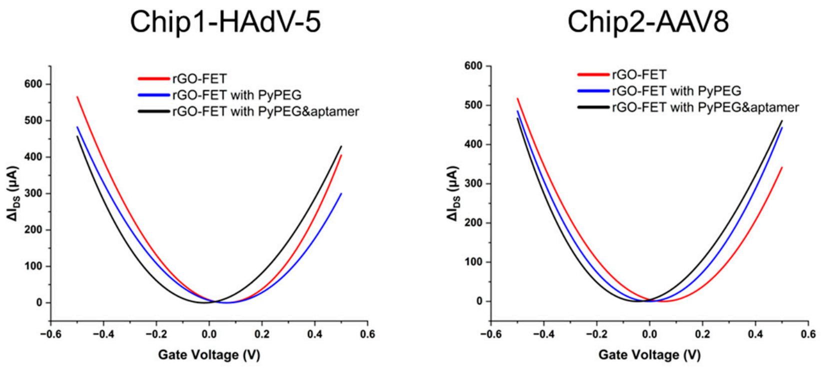

Figure S2. Layer-by-layer functionalization of reduced graphene chip surfaces using PyPEG linkers and subsequently EDC/NHS-mediated coupling of NH_2-labeled anti-HAdV-5 aptamer library for each chip used for the experiments shown in Figure 6.

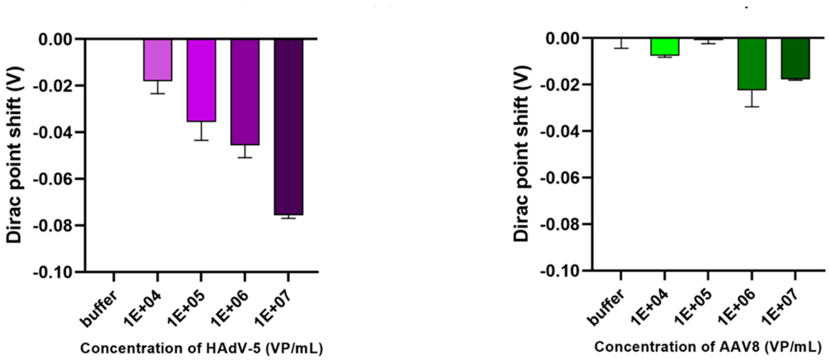

Figure S3. Dirac point shifts (Δ_{Dirac}) resulting from $I_{DS}V_G$ transfer curves measured in Figure 6. Error bars represent standard deviations.
